# The Intriguing Pattern of Nontuberculous Mycobacteria in Bulgaria and Description of *Mycobacterium bulgaricum* sp. nov.

**DOI:** 10.3390/ijms251910434

**Published:** 2024-09-27

**Authors:** Danila Zimenkov, Yuliana Atanasova, Anastasia Ushtanit, Stanislava Yordanova, Ana Baykova, Marina Filippova, Uliana Semenova, Igor Mokrousov, Elizabeta Bachiyska

**Affiliations:** 1 Center for Precision Genome Editing and Genetic Technologies for Biomedicine, Engelhardt Institute of Molecular Biology, Russian Academy of Sciences, 119991 Moscow, Russia; ushtanit@mail.ru (A.U.); mafilippova@mail.ru (M.F.); chichevna@yandex.ru (U.S.); 2 National Reference Laboratory of Tuberculosis, Department of Microbiology, National Center of Infectious and Parasitic Diseases, 44A General Nikolai Stoletov Boulevard, 1233 Sofia, Bulgaria; s.yordanova@ncipd.org (S.Y.); tb_nrl@abv.bg (A.B.); elizabetbatchiiska@abv.bg (E.B.); 3 Laboratory of Molecular Epidemiology and Evolutionary Genetics, St. Petersburg Pasteur Institute, 197101 St. Petersburg, Russia; imokrousov@mail.ru

**Keywords:** nontuberculous mycobacteria, infection disease, diagnostics, whole-genome sequencing

## Abstract

We investigated the rise of nontuberculous mycobacteria (NTM) infections in Bulgaria, focusing on species identification and distribution from 2018 to 2022. Utilizing advanced diagnostic tools, including the Hain Mycobacterium CM/AS method, Myco-biochip assay, and whole-genome sequencing, the study identifies and characterizes a diverse range of *Mycobacterium* species from clinical samples. While *M. avium*, *M. gordonae*, *M. fortuitum*, and *M. chelonae* were dominating, a number of rare species were also found. They include such species as *M. marseillense* and *M. celatum*. Moreover, the noticeable prevalence of *M. terrae* complex species missed by conventional testing was observed. We identified a rare species, highly homologous to previously described strains from Japan; based on genome–genome distance data, we propose its reannotation as a new species. Further, a novel species was identified, which is significantly distinct from its closest neighbor, *M. iranicum,* with ANI = 87.18%. Based on the SeqCode procedure, we propose to name this new species *Mycobacterium bulgaricum* sp. nov. Dynamic changes in NTM species prevalence in Bulgaria observed from 2011 to 2022 highlight the emergence of new species and variations tied to environmental and demographic factors. This underscores the importance of accurate species identification and genotyping for understanding NTM epidemiology, informing public health strategies, and enhancing diagnostic accuracy and treatment protocols.

## 1. Introduction

The genus *Mycobacterium* includes an ever-increasing number of species with contrasting phylogeographic and biological features. The global phylogenetic tree of *Mycobacterium* consists of more than 200 different species [1,2], which present significant variability in terms of pathogenic properties and clinical manifestations of infections [3]. Mycobacteria inhabit both the natural environment and public infrastructure, causing the infection from direct contact or indirect, like aerosols and dust [4,5].

As the incidence of tuberculosis has decreased globally over the past decade, there has been an increase in disease caused by nontuberculous mycobacteria (NTM) [6]. The global rise of NTM infections across the world can be attributed to a combination of enhanced diagnostic capabilities and a growth in the population vulnerable to such infections [7,8]. The incidence rate of NTMs across the world is varying in a wide range, with the highest rates reported in 2010s being more than 20 cases per 100,000 population in the USA [9], Canada [10], Taiwan [11], and Australia [12]. In other regions the incidence rate is lower; in the Netherlands, the rate is estimated between 2–5 cases per 100,000 [8], and 4.8 cases were reported in Korea [13].

The incidence and severity of NTM diseases rapidly rise in the elderly population. Thus, in people older than 65 years, the incidence is estimated between 47 per 100,000 in 2007 [14] and more than 100 cases for people older than 80 years [9]. For comparison, the tuberculosis incidence rate (new cases per 100,000 population per year) is estimated to have increased to 133 (95% UI: 124–143) in 2022 globally. In the European region, the reverse trend was observed—the incidence rate dropped from 42 in 2010 to 25 in 2022 [15].

Tuberculosis and NTM diseases have similar clinical manifestations, which hardens the specific diagnosis and demands the use of the advanced molecular diagnostic tests [16]. In New Delhi, India, NTM diseases were detected in 17.6% of the suspected pulmonary multiple drug resistant’tuberculosis cases and 12.4% of suspected extra pulmonary tuberculosis cases [17]. The prevalence of NTM was 9.7% among tuberculosis suspected in 2012–2013 in Tanzania, with tuberculosis prevalence of 217 and 244 per 100,000 in urban and rural settings, respectively [18]. Differentiation of NTM disease and tuberculosis with identification of particular species of *Mycobacteria* is thus a main task for laboratory diagnostics [19].

Wengenack et al. [20] recently suggested complementing the phylogenetic classification of NTM species by a large-scale subdivision into complexes of the related species with clinical significance. By definition, this scheme does not include non-pathogenic NTM species. The species most frequently isolated from clinical samples belong to *M. avium*, *M. chelonae-abscessus*, and *M. kansasii* complexes, thus supposed to be the most pathogenically significant mycobacteria. However, the spectra of the identified species causing the disease are steadily growing. Definition of a clinical relevance of particular species is a complex task [21], and each independent description is of great importance, allowing the estimation of the disease cause and progression.

The distribution of NTM species is not uniform and varies considerably across different countries and regions, suggesting that local environmental factors and differences in clinical practices might influence the prevalence of particular species [22,23]. In a recent systematic study covering the published reports from regions all around the world, the prevalence of species from the *M. avium* complex, mainly *M. avium* itself and *M. intracellulare*, was demonstrated [24]. However, in particular settings, other mycobacterial species were identified as the most frequent: *M. abscessus* in Taiwan and Indonesia [25,26], *M. kansassii* in China and Spain [27,28,29]. Nevertheless, the global trend of an overall increase in NTM worldwide for both infection and disease is keeping [24]. In this sense, Bulgaria being located in the European region is no exception, while the published reports from this country in English are scarce [30,31]. The NTM burden was recently estimated for the first time by Atanasova based on different kinds of criteria. Applying the possible and probable disease criteria (microbiologically confirmed clinical pulmonary disease), the annual incidence is 1 per 100,000, and applying only the microbiological criteria (probable disease)—0.23 per 100,000 [32]. 

A total of 183 patients with isolated NTM isolates were reported in Bulgaria in 2018–2020, and along with dominant species, there were minor species that may also have medical significance. Some of them remained ‘non-identified,’ or identified only to genus level due to the ‘blindness’ of the most widely used commercial molecular tests. Non-identified *Mycobacterium* spp. in Bulgaria accounted for 8.2% of samples [30]. 

Importantly, rarely isolated species should not be considered contaminants and thus not clinically relevant by default. The number of reports that certain mycobacterial species have clinical significance and most of them are difficult to identify by biochemical and/or routine molecular tests is steadily growing [33,34,35,36,37]. In this sense, the use of molecular methods based on genomic signatures based on single or multiple loci sequencing or whole genome data is the most comprehensive approach to studying such rare NTM species [38,39].

At present, there are various methods used for NTM species identification [40,41]. Some of them have been implemented and widely used, like Hain Lifescience Genotype CM/AS assays (HAIN) [42], while others based on analysis of single or several genes were published in research articles [43,44,45,46,47,48]. While the HAIN method is a line-probe macroarray methodology based on the analysis of slow-evolving 23S rRNA gene fragments, other approaches, targeting the more variable fragments of *gyrA*, *gyrB*, *rpoB*, *hsp65*, *secA1* genes, and ITS region between 16S and 23S rRNA genes, provide a more comprehensive approach that still requires careful optimization for sensitivity, specificity, and overall performance. The Biochip method, based on hybridization with the panel of oligonucleotide probes, was previously used to detect genetic determinants of drug resistance and phylogenetic lineages and clusters of *M. tuberculosis* and for identification of NTM species [49,50].

Specific data on the isolation of NTM in Bulgaria highlights the urgent need for detailed characterization of NTM species in the country to improve diagnostic and treatment strategies. The aim of the present study was an evaluation of the developed Myco-biochip assay on Bulgarian samples, in particular those non-identified by the currently used molecular methods, and a more precise characterization of the NTM species structure in Bulgaria.

## 2. Results and Discussion

### 2.1. Nontuberculous Mycobacteria Profile in Bulgaria

Bulgaria is a European and, more specifically, Balkan country with population size 6,385,500 and population density 63/km^2^ (https://en.wikipedia.org/wiki/Bulgaria, accessed on 22 September 2024). The tuberculosis laboratory network of Bulgaria consists of 30 laboratories for culture-based diagnostics, including NRL, and 29 regional laboratories. In 2018 to 2022, a total of 117,791 clinical samples were processed from 62,565 patients suspected of having tuberculosis or for differential diagnostics of other pulmonary diseases. Sputum, bronchoalveolar lavage, gastric washes (in children), and biopsy materials were collected in the regional pulmonary hospitals or wards performing the diagnosis, treatment, and control of tuberculosis in Bulgaria. The species identification of NTM was performed only in the National Reference Laboratory of Tuberculosis from pure culture.

In 2018–2022, 290 patients with presumably NTM disease were identified. The isolates were identified at the species level by Hain Lifescience Genotype CM and AS assays. The top frequent NTMs were *M. avium* and *M. gordonae* (Table 1), accounting for about 35% and 26%, respectively. In total, 18 different species were detected, while 5.2% of isolates were identified only to the genus level.

Further, a selection of the 74 identified isolates from 2022 and all available non-identified isolates from 2018–2022 was subjected to the Myco-biochip analysis. We aimed to assess the concordance of the two methods (Hain assay and Myco-biochip) and to see if the Hain-non-identified isolates can be assigned to the particular species by the biochip-based assay. As a reference method, *gyrB* and 16S rRNA gene sequencing and, in some cases, WGS analysis were performed.

Thus, for further biochip analysis and gene sequencing, we selected 48 isolates from 48 patients. Of them, 40 DNA identified at the species level by Hain assay are from 2022 (54% of 74 identified NTM isolates in 2022) and belonged to the following species: *M. avium*—10, *M. intracellulare*—6, *M. gordonae*—4, *M. fortuitum*—3, *M. lentiflavum*—3, *M. chelonae*—2, *M. chimaera*—2, *M. mucogenicum*—2, *M. abscessus*—1, *M. celatum*—1, *M. kansasii*—1, *M. marinum*—1, *M. scrofulaceum*—1, *M. simiae*—1, and *M. xenopi*—1. The species names are given according to the Hain assay results. Also, we studied all available isolates identified only to a genus level (*n* = 8) from the 2018–2022 period.

As a result, the species identity of isolates from the 2022 collection was concordant with Hain and Biochip for most isolates (Supplementary Table S1). Based on Hain, forty of forty-eight isolates were assigned to various *Mycobacterium* species, and eight were non-identified. For those 40 identified by Hain at species level, the results were discrepant with the biochip assay for seven samples. Use of *gyrB* sequencing confirmed biochip results in seven samples, and Hain result in one sample. In particular, Hain-determined *M. gordonae* were assigned to *M. paragordonae* (Supplementary Table S1). 

Two molecular methods—Hain Mycobacterium CM/AS and Myco-biochip—share the common settings of amplification of the genomic locus and hybridization with species-specific probes. Hain tests utilize the *rrl* gene (coding for 23S rRNA) fragment, and biochip uses the *gyrB* gene fragment. The biochip identification was based on the similarity of hybridization pictures of perfectly matched and mismatched interactions with probes. Due to partial homology of sequences in different species, some uncharacterized sequences could produce a specific hybridization pattern due to imperfect binding to mismatched sequences [49]. Further sequencing of an isolate and assigning of the profile to species expands the sensitivity of the biochip test system. Thus, we identified two novel profiles: (i) one for sample #36 and (ii) another common to samples #43 and #44, which is discussed in detail below.

At the higher taxonomy level of *Mycobacterium* complexes [20], comparison of two molecular methods showed that 39 of 40 isolates identified at the species level were concordantly assigned by the two methods to the same complex according to the recent nomenclature of common nontuberculous mycobacteria [8]. One exception was isolate #18 that was identified as *M. scrofulaceum* (*M. scrofulaceum* complex) by the Hain tests and *M. mucogenicum* (*M. mucogenicum* complex) by the biochip analysis.

Sequencing of the *gyrB* fragment revealed a homology with *M. mucogenicum* strain 1199456.5 isolated in Mozambique (GCF_001665375.1). There were only 3 mismatches per 674 base-pair fragment. However, this strain should be reannotated as *M. phocaicum* [51] due to its close homology to the sequences of the type strains JCM 15301 (GCF_010731115.1) and DSM 45104 (GCF_005670655.1) with ANI = 95.5% (ANI—average nucleotide identity). The distance to the genome of *M. mucogenicum* [52] strain DSM 44124 (GCF_005670685.2) constitutes only 92.3%. 

Similarly to sample #18, two more isolates identified as *M. mucogenicum* by both Hain and biochip assays should be reannotated as *M. phocaicum* based on published genome sequences and ANI criteria for species delineation. Isolate #17 had a *gyrB* fragment sequence identical to genome GCF_026501155.1, representing isolate 15IE37 obtained from the sputum of a patient in Ireland (Flanagan et al., unpublished; https://www.ncbi.nlm.nih.gov/nuccore/JAPMJT000000000, accessed on 1 August 2024). While the isolate was also annotated as *M. mucogenicum*, its sequence is also closer to the sequence of the type strain *M. phocaicum* than to the type strain *M. mucogenicum*—95.6 and 91.9% ANI, respectively. Another isolate #40 had a sequence close to the genomic sequence GCF_003851485.1 of the *M.* sp. NCC-Tsukiji [53] with 20 mismatches per 674 bases. It supposedly belongs to *M. phocaicum* species since the ANI distance between this genome and the above-mentioned *M. phocaicum* (GCF_010731115.1) constituted 95.2%. The distance of the obtained *gyrB* fragment of isolate #40 to *M. phocaicum* constituted 23 and 31 mismatches to the *M. mucogenicum* sequences, thus we annotated this isolate also as *M. phocaicum*. 

The final list of the identified *Mycobacterium* complexes and species and number of isolates per each is shown in Table 2. Biochip and *gyrB* were jointly more performing and identified eight Hain-non-identified isolates at the species level, further verified by target sequencing and WGS. 

### 2.2. NTM Species Distribution in Age- and Gender-Stratified Groups

The studied NTM isolates were obtained from patients with a median age of 62.9 ± 15.0 years old. This is similar to the Bulgarian study of all isolates available in 2018–2021, when the most vulnerable group were patients >65 years old (41%) [30,31]. Age over 65, as well as female gender and low body mass index, are the main predispositions for NTM disease globally [54]. General population aging in Bulgaria [55] and in developed countries overall [56] is thus one of the main factors responsible for NTM incidence rise.

The distribution of complexes in all and in age and gender subgroups of Bulgaria (all 48 samples) shows the following: There was some difference in species distribution between male and female groups (Table 3). The larger female group showed a greater number of species, although it was relatively dominated by *M. avium* complex isolates. Only some minor species were detected in either males (all 3 *M. fortuitum* complex isolates) or females (both *M. abscessus* isolates), but most species were identified in both gender groups. *M. avium* complex isolates were more prevalent in the female group but non-significantly (13/29 vs. 4/17, *p* = 0.2). The second largest complex (*M. terrae*) was detected in 3 patients in each gender group.

Regarding species distribution in different age groups, Table 4 shows information on all 46 isolates with age information available. Since most patients were >59 years old, such a comparison cannot be meaningful. We can only note that a single *M. marinum* isolate was a 47-year-old male and not from sputum but from wound secretion (not surprising for this species). Otherwise, all other identified species were present in the age group >59 years old. Only two species complexes—*M. avium* complex and *M. terrae* complex—were present in all age groups.

Two patients infected with *M. abscessus* (the same year 2022, isolates 1_22 and 2_22) were both from Sofia, female, 67 and 70 years old, but we did not find any information that can link these patients directly.

### 2.3. M. terrae Complex in Bulgaria

Geographic distribution of the isolates/complexes in provinces across Bulgaria showed that most of the studied NTM isolates were from Sofia (Table 5). The largest collection from Sofia was the most diverse on the whole. The largest group of *M. avium* complex isolates was detected in almost all locations. However, all six *M. terrae* isolates were not from the capital city of Sofia but from other and distant locations—three from Varna, others from cities in different parts of the country. Interestingly, *M. terrae* was found in Bulgaria in 2018–2020, but not in 2021–2022.

The clear dominance of the *M. terrae* complex isolates in the previously non-identified group (six of eight isolates) is remarkable. Slow-growing mycobacteria from the *M. terrae* complex were supposed to be rare pathogens, usually found in cases of tenosynovitis and osteomyelitis [57,58]. Cases of lung infections were also described [59,60]. But the rare descriptions of single cases allowed us to consider it as a colonizing agent in cases of identification in clinical samples [61]. While we did not identify the *M. terrae* species itself, other members of the complex were identified by the combined analysis using Biochip and sequencing: *M. kumamotonense*—three samples, *M. hiberniae*—two samples, and *M.* sp. GF74—one sample. The identification of the latter based on whole-genome sequencing (WGS) is described in detail below.

*M. terrae* complex member *M. kumamotonense* [62] is rarely isolated from patients, and there are only sporadic cases across the world known up to date [18,63]. The highest prevalence was recorded only in Ankara, Turkey, with six cases from nineteen clinical specimens [64]. A close and long-term human interaction of Bulgaria and Turkey took place in the historical and recent past [65,66,67,68]. An ongoing human exchange and travel is considerable, in particular, due to the visible proportion of Bulgarian citizens of Turkish ethnicity living in Turkey and visiting Bulgaria [69]. Therefore, it is tempting to speculate that this situation is underlying the historically distant and endemic presence of *M. terrae* in both Bulgaria and Turkey and distinguishes these countries from their neighbors.

Nevertheless, *M. kumamotonense* could cause lung and soft tissue infections [70] in immunocompromised [71] and immunocompetent persons [21,72,73], and thus should not be considered as a colonizing agent and demands attention upon identification in clinical samples. In our study, two patients (both male, from Varna, the same year 2020) were infected with *M. kumamotonense* (isolates 46 and 47), but we did not find any information that can link these patients directly. We were not able to trace contacts between them or shared use of urban areas.

While the *M. kumamotonense* was identified by biochip analysis, two other samples #43 and #44 produced identical novel-specific biochip hybridization profiles. Further sequencing of the *gyrB* fragment allowed its annotation as *M. engbaeki* [74] since both 306 bp fragments were identical to the genomic sequence of strain ATCC 27353 (GCF_002101585.1). This species is a close relative to *M. hiberniae* [75], with ANI between them slightly above the 95%. However, whether they constitute one species is still an open question [76]. Both species are low pathogenic, mostly isolated from the environment and animals [75,77]. While there are reports on identification of *M. engbaeki* [74,78], *M. hiberniae* was not found in clinical samples. However, it could be due to problems with correct identification of the species both by widely used Hain CM/AS tests and target sequencing [18]. 

In addition, *M. hiberniae* has a rose-pink pigmentation, which is unique for *Mycobacterium* [75]. Our isolates were strictly yellow on Middlebrook 7H9-OADC agar plates, adding to the existence of separate species.

### 2.4. Description of Mycobacterium bulgaricum sp. nov.

One previously non-identified isolate #49 was initially assigned to the genus *Mycobacterium* by the Hain test and to *M. iranicum* by the biochip assay. The biochip identification was based on the analysis of the previously described clinical isolate MC-434, obtained from the sputum of the patient in Moscow, Russia [49]. The sequence of the *gyrB* gene fragment was also identical to the sequence of the isolate MC-434 (NCBI accession no. KJ725343.1). The strain was annotated as *M. iranicum* then based on the sequences of 16S and ITS fragments. The *gyrB* fragment alignment revealed only a weak homology to the *M. smegmatis* sequence with a similarity of about 91%.

The strain #49 was isolated from the sputum of a 68-year-old female in Sofia. The whole-genome sequencing was performed for this isolate. The draft genome was reconstructed from the short reads with average coverage of 230×, resulting in 85 contigs of 6,074,737 bp. The genome sequence and the sequence of the 16S rRNA gene were used for the taxonomy pipeline Protologger, recommended for delineating the novel taxonomic units [79]. The 16S rRNA was 99.17% identical to *M. iranicum* (HQ009482), while the genomic sequence was novel, with ANI = 87.18% to the closest genome of *M. iranicum*, i.e., well below the species identity threshold of 95%.

The analysis of the genome using the ‘wgs’ database of NCBI confirmed the homology with genomes of *M. iranicum* strains DSM 45541 (GCF_002101705.1), UM_TJL (GCF_000455165.1), and SBH312 (GCF_026805495.1) with very similar ANI values of about 87.12%. *Mycobacterium iranicum* is a clinically relevant species associated with pulmonary and cutaneous infections [80,81,82]. The genome of isolate #49 is also similar to the *Mycobacterium* strain BiH015 isolated from the soil in Vietnam with ANI = 87.02%. The obtained ANI values are below the approved borderline for species delineation of 95% [83], accounting for the obtained genome sequence contamination of 0.18% estimated by Protologger. We thus suppose that #49 is a representative of the novel species inside the proposed *M. aurum* complex (Zimenkov, submitted [84]) together with *M. aurum*, *M. vaccae*, *M. parafortuitum*, *M. gilvum*, *M. iranicum*, *M. austriafricanum,* and other still unnamed species.

The raw data and the assembled genomes were deposited at the NCBI, checked for contamination, sequence depth, and closest species using the Protologger pipeline. The description is thus following the Code of Nomenclature of Prokaryotes Described from Sequence Data (SeqCode) [85] and in compliance with the requirement of the International Code of Nomenclature of Prokaryotes (2022 Revision) [86]. We propose to name this novel species *Mycobacterium bulgaricum* sp. nov. to indicate Bulgaria as the first place of isolation.

### 2.5. Novel Isolates of Two Orphaned Species Represented by Strains GF74 and GF28

Besides the above novel species, we also identified two isolates that belong to the rare orphaned species without deposited type strains and correct species names.

The first isolate #45 was recovered from the sputum of the 78-year-old male patient from Varna. It was identified to the genus level by the Hain test and as *M. senuense* by the biochip analysis. Similarly to the above-described isolate #49, the identification was based on the previously studied clinical isolate pK-82 from Moscow, Russia [49]. That isolate had the sequence of a *gyrB* fragment with low homology to known sequences (KJ725322.1), and the annotation was based on the homology of the 16 rRNA gene. The sequence had one mismatch per 453 bases compared with *M. senuense* strain 05-832 (NR_043905)—the type strain of the specie [87]. The obtained sequence of *gyrB* of #45 isolate was similar to that of pK-82 with seven mismatches. However, compared with the whole genome sequence of *M. senuense* published in 2019 [39], *gyrB* fragments had 17 mismatches per 306 bases for both Russian pK-82 and Bulgarian #45. A more similar fragment was found in the whole genome sequence of isolate GF74, also annotated as *M. senuense* [88]. There were only nine and eight mismatches for pW045 and pK-82, respectively. While both genomes of *M. senuense* GF74 and the type *M. senuense* 05-832 share the same *rrs* sequence, the annotation of GF74 should be reconsidered since the ANI value between them is only 91.86%, which is below the breakpoint value.

For more precise species identification of Bulgarian isolate #45, we performed its whole genome sequencing. The assembled genome had low contamination levels and comprised 4,483,975 bp, while the estimated length was 5,236,316 bp. The closest genome was that of Japanese strain GF74 with ANI = 97.58%, as expected. This value is higher than the species breakpoint delineation value and close to that used for subspecies delineation. Thus, we conclude that Bulgarian isolate #45 belongs to the same species as previously described strain GF74, and both differ from *M. senuense*. Such an independent description confirms the existence of a novel species inside the *M. terrae* complex. However, the isolation of this mycobacteria does not reflect its clinical significance since the Bulgarian patient did not have significant pathology signs and thus does not fit the diagnosis criteria.

The second interesting Bulgarian isolate #36 was recovered from the sputum of the 69-year-old female patient from Plovdiv. It was identified as *M. intracellulare* by the Hain test and as a novel, unknown profile by biochip. The sequence of the *gyrB* gene fragment was identical to the fragment of the genome of strain GF28, annotated as *M. colombiense*. Strain GF28 was identified in the same Japanese study along with the above-mentioned strain GF74 [88]. Strain GF28 differs from *M. colombiense* [89], represented by the sequences of the type strains CECT 3035 (GCF_002105755.1) and CSUR P297 (GCF_900161855.1), with an ANI value of 89.7%. However, it definitely belongs to the *M. avium* complex as well as *M. intracellulare* and *M. colombiense*.

Mycobacterial strains GF74 and GF28 were isolated from mud at a swine farm in the Tokai area of Japan and were initially identified to the species level by analyzing the 16S rRNA, *hsp65*, and *rpoB* genes [88]. However, separate loci could lead to misinterpretation since identity borderline values should be established in a genus-wide manner. Therefore, the WGS-based approach for delineating species is the current gold standard. Ito et al. pointed out that strain GF74 is 93.12% identical to *Mycobacterium* sp. strain JDM601, and strain GF28 is 86.73% identical to *Mycobacterium indicus pranii* [71]; the latter, to be precise, is not a separate species but a part of *M. intracellulare*, with 99.7% genome identity. Both values are below the 95% border, confirming the standalone nature of the found species. Therefore, we suggest that our description of new strains genomically similar to GF28 and GF74 confirms the existence of two novel species inside the *M. avium* and *M. terrae* complexes, respectively, expanding our knowledge on the global mycobacterial diversity.

### 2.6. Comparison with Longitudinal Data on NTM Species Distribution in Bulgaria

We compared results on the 2022 collection with the earlier data on the NTM species in Bulgaria for 2011–2017 [32] and 2018–2021 [30] (Table 1).

Few observations can be made about dynamic changes in the NTM species prevalence from 2011 to 2022. There is a steady increase of the definitely pathogenic species of the *M. avium* complex, from 16.1% in 2011–2017 to 44.6% in 2022 (*p* < 0.0001), and a less pronounced increase in the *M. gordonae* complex, from 17.5% to 24.3% (*p* = 0.15), although it should be noted that the latter are usual contaminants of colonizers. There was some increase in the rare *M. xenopi* isolates.

In contrast, some species showed a decrease in isolation: *M. lentiflavum* (from 15.2% to 5.4%; *p* = 0.03), *M. fortuitum* (from 10.6% to 5.4%; *p* = 0.17), and *M. chelonae* (from 8% to 2.7%; *p* = 0.11). Some globally important species, first of all, *M. kansasii*, being rare enough at 2.9% in 2011–2017, have almost disappeared in 2018–2022. Similarly, the rare pathogen *M. peregrinum* was nearly completely lost recently (Table 1). *M. abscessus* was identified in similar 2–4% during all observation periods.

### 2.7. European and Regional Context of NTM in Bulgaria and Neighbors

Comparison of data on Bulgaria, including isolates re-identified in this study, with available data on the Bulgarian neighbors (Serbia, Turkey, and Greece) is shown in Table 6. In general, the data are based on the information on the main species detected by the Hain assay [90,91,92].

*M. avium* complex was the most prevalent in all 4 countries but was especially dominant in Greece (64.8% compared with 16.1% in Bulgaria in approximately the same period (*p* < 0.0001). For certain species, contrasting prevalence rates were observed between some countries, although different periods of sampling should be taken into consideration.

*M. terrae* was specifically visible in the studied Bulgarian sample; however, species of the *M. terrae* complex are not identified by Hain tests [42] and thus were not estimated in other studies. Thus, there is no information about the prevalence of these strains in Serbia, Turkey, and Greece in the cited articles. However, in another study from Turkey, *M. terrae* complex isolates were described [64]. See subsection on the *M. terrae* complex above.

*M. abscessus* was more prevalent in Serbia and Turkey compared with Bulgaria (*p* = 0.002 and 0.01, resp.) and Greece (*p* = 0.06 and 0.1, resp.); in the latter case, the small sample size could be the reason for non-significant *p* values. *M. xenopi* was most prevalent in Serbia (23%) compared with 1% in Bulgaria (*p* < 0.0001) and 2.8% in Greece (*p* = 0.002). *M. mucogenicum* was detected only in Bulgaria at 2.5% but was not described in its neighbors. Interestingly, *M. gordonae* was especially prevalent in Turkey and was identified mainly as a contaminant.

The contrasting prevalence rates of some NTM species in the neighboring countries may hypothetically point to the difference in water supply systems. For example, *M. xenopi* is dominant only in Serbia; *M. kansasii* is extremely rare only in Bulgaria. In the USA, the major source of NTM and *M. avium* is in-house piped water [93]. On the other hand, free human transborder movement could alter the NTM spectra due to the gap between infection and disease detection.

Dynamically changing environments, natural and human-engineered, can facilitate the circulation of certain NTM species (more resistant to such environments) but lead to the decline of other less resistant NTM species. Falkinham et al. measured the desiccation susceptibility of different NTM strains adhering to stainless steel and found that survival of the main species of *M. avium* complex was much higher compared with *M. abscessus* complex (0.6%) and *M. chelonae* (<0.02%) [93].

The phylogeography pattern of *M. gordonae* may be more difficult to interpret since this species is in general not clinically relevant, and this may explain its absence in clinical cases in Greece and Turkey (while it was dominant as a contaminant in Turkey). In this sense, isolation of *M. gordonae* at 25% in Bulgaria and 5% in Serbia is likely not clinically relevant. As noted by Bachiyska et al. [30] about the survey of NTM in 2018–2021 in Bulgaria, only 25% of patients with NTM isolates met the American Thoracic Society (ATS) criteria for microbiologically confirmed pulmonary disease.

*M. kansasii* previously annotated as *M. kansasii* subtype 1 [94], cause infections in humans independently of the immune status of the host [95], and thus is one of the species with the highest clinical significance among nontuberculous mycobacteria. Slow-growing *M. kansasii* is the second most common cause of pulmonary NTM disease in Europe [23]. However, the prevalence of *M. kansasii* in Bulgaria is unexpectedly low at ~2% in the 2011–2022 collection. There is also a tendency to its decline, since only one isolate was found in 2018–2022. The significant variation of the overall frequencies of *M. kansasii* across different countries in Europe was observed between 0% and 36% [79]. The highest prevalence was in Poland and Slovakia with 35% and 36%, but in the neighboring Germany and Austria the prevalence was as low as 6% and 4%, respectively. There were no data from Bulgaria or any of its neighbors in the cited study [96], however, in Serbia it was 17.6% [90]. The isolation of *M. kansasii* is related to mining activities, industrialization, urbanization [96], and particularly with water supply systems [97]. Unlike other nontuberculous mycobacteria, *M. kansasii* is not readily isolated from environmental sources [97]. Two recent studies confirmed the city water distribution systems as the main paths of infections in Australia [98] and the Czech Republic [99].

## 3. Materials and Methods

### 3.1. Mycobacterium Species Identification and Microbiology Methods

The species identification of NTM was performed in National Reference Laboratory of Tuberculosis (NRL TB), National Center of Infectious and Parasitic Diseases (NCIPD), Sofia, Bulgaria. The quality of the laboratory’s diagnostic work is externally controlled and certified by the Institute for Standardization and Documentation of Medical Laboratories INSTAND e. V., Dusseldorf, Germany.

Patients suspected of having tuberculosis or included in the differential diagnostic plan for detection of other pulmonary diseases were enrolled in the study. Samples (sputum, bronchoalveolar lavage, gastric washes (in children), and biopsy materials) were collected in the regional pulmonary hospitals or wards performing the diagnosis, treatment, and control of tuberculosis in Bulgaria. A smear microscopy for AFB (Ziehl–Neelsen) from the specimens was performed. The strains were isolated using solid (Loewenstein–Jensen) and liquid media using BACTEC MGIT 960 System (Becton Dickinson Microbiology Systems, Cockeysville, MD, USA) according to the relevant standard operating procedures of the national and international guidelines [30]. After a negative immunochromatographic test (SDTB Ag MPT64 Rapid, Standard Diagnostics, Republic of Korea), cultures were transferred to NRL TB, NCIPD, for species identification.

It should be noted that culturing of NTM isolates from clinical samples does not always mean NTM pulmonary disease. The diagnostic criteria of NTM pulmonary disease—clinical, radiographic, and microbiologic criteria—are equally important, according to the official clinical practice guidelines of ATS/ERS/ESCMID/IDSA [100]. The microbiologic criteria include positive culture results from at least two separate expectorated sputum samples or positive culture results from at least one bronchial wash or lavage, or transbronchial or other lung biopsy [30]. In the present study, we included all mycobacterial isolates recovered from clinical specimens.

DNA was typed using GenoType Mycobacterium CM and AS kits (HAIN Lifescience, Nehren, Germany), which are based on DNA strip technology: PCR amplification of 23S rRNA gene, reverse hybridization with specific probes immobilized on membrane strips.

### 3.2. Biochip Assay and Sanger Sequencing

Since ribosomal operon is not a reliable phylogenetic marker due to recombination, horizontal transfer, and low mutation rate [101,102], other genes are used for species delineation [17,46]. The *gyrB* locus was selected based on previous studies on mycobacterial species identification [103,104] and our own studies, which confirmed the specificity of *gyrB*. Its diversity even allows the subspecies identification, as shown for *M. avium* [105]. 

The *gyrB*-based biochip test for *Mycobacteria* species identification was described previously [49]. The first stage of the procedure includes asymmetric PCR amplification of the ~300 base *gyrB* fragment with simultaneous fluorescence labeling by sulfo-Cyanine5 dUTP conjugate (Lumiprobe RUS Ltd., Moscow, Russian Federation). For amplification of the fragment for sequencing, the following primers were used: Forward 5′-CCTTCGCSAACACSATCAACAC-3′, Reverse 5′-CGTCIACGTCSGCRTCGGCCAT-3′, and alternative Forward-2 5′-CCCAAGTCCGCCGAGGA-3′.

The resulting predominantly single-stranded amplicon was hybridized with gel-immobilized oligonucleotide probes arranged on plastic plate. Fluorescent microscope (Biochip-IMB Ltd., Moscow, Russian Federation) was used for registration of hybridized product after washing with warm water. Species-specific hybridization profiles were compared with the local database of profiles obtained using the reference isolates. The specificity of novel profile was based on a number of significant probes with high signals compared with background and low correlation with known profiles. In such cases, target sequencing of *gyrB* gene fragments was used for species identification.

### 3.3. Whole-Genome Sequencing

Strains for whole-genome sequencing were cultured on Lowenstein–Jensen media for approximately 4 weeks at 37 °C and then heat-inactivated. Genomic DNA was extracted using the Gentra Puregene Yeast/Bact. Kit (cat no. 158567, QIAGEN, Venlo, The Netherlands).

DNA libraries were prepared using the MGIEasy Universal DNALibrary Prep Set (MGI Tech, Shenzhen, China) according to the manufacturer’s protocol. The concentrations of DNA libraries were measured using Qubit Flex (Life Technologies, Carlsbad, CA, USA) with the dsDNA HS AssayKit (Invitrogen, Waltham, MA, USA). The quality of the prepared libraries was assessed using Bioanalyzer 2100 with the High Sensitivity DNA kit (Agilent Technologies, Santa Clara, CA, USA). The enriched library pools were further circularized and sequenced via paired end sequencing using DNBSEQ-G400 with the DNBSEQ-G400RS High-throughput Sequencing Set PE100 (MGI Tech, Shenzhen, China).

Genomes were further assembled to contigs from Fastq files using Shovill (https://github.com/tseemann/shovill, accessed on 30 July 2024) at the Galaxy web platform public server (https://usegalaxy.org, accessed on 30 July 2024) [106]. Genome data were deposited to NCBI SRA and Genome databases (PRJNA1138261). 

Genome–genome distances were estimated using the average nucleotide identity (ANI) value by FastANI approach [107]. Genomes of presumably novel species were analyzed using the Protologger [79] via online portal (https://protologger.bi.denbi.de/, accessed on 1 August 2024). The formal description of new species was in compliance with the requirement of the International Code of Nomenclature of Prokaryotes (2022 Revision) [86].

### 3.4. Statistical Analysis

Statistical analysis was performed using MedCalc online tool (https://www.medcalc.org/calc/odds_ratio.php, accessed on 22 September 2024). A chi-square test was used to detect any significant difference between the two groups. Yates corrected χ2, and *p*-values were calculated with a 95% confidence interval (CI) around the mean. The significance threshold was set at *p* = 0.05.

## 4. Conclusions

NTM are highly heterogeneous in pathogenicity, clinical features, and geographic pattern, whereas accurate species identification is essential in determining etiotropic therapy. This emphasizes the importance of the correct identification of the NTM isolates to the species level and understanding their epidemiology. Accurate species identification has crucial implications for patient treatment, emphasizing the need for the development of precise diagnostics and continuous surveillance strategies for NTM infections. Molecular high-density assays like Biochip rely on robust phylogenetic markers other than rRNA gene fragments and present such a reliable tool for NTM species identification. 

This study allowed for precise and refined identification and characterization of nontuberculous mycobacteria species in Bulgaria by using a combination of the Myco-biochip assay and *gyrB* analysis verified by WGS. The NTM population structure in Bulgaria is dominated by *M. avium* complex, whose prevalence has been increasing in the last decade. A clinically important NTM species, *M. kansasii,* is extremely rare in Bulgaria compared with its neighbors. We demonstrated the existence of three new NTM species, including one that we tentatively named *Mycobacterium bulgaricum* sp. nov. However, this species is unlikely to be associated with disease. We revealed an intriguing dominance of the *M. terrae* complex isolates among previously non-identified samples.

A speculative yet possible explanation of the changing prevalence and diversity of different NTM species in time and/or in space may at least partly be due to their internal population structure at the subspecies level. In other words, in response to changing human-determined or natural environments, different strains within different species can demonstrate higher or lower capacity for adaptation. In this view, high-resolution genotyping of the medically important NTM strains/species could provide scientifically interesting and practically useful clues in this regard. Placed within the geographic and temporal context, our findings underscore the dynamic nature of NTM species prevalence, influenced by as yet unclear regional and environmental factors that warrant further study.

## Figures and Tables

**Table 1:** Detection of nontuberculous mycobacteria in Bulgaria in different year periods.

	Bulgaria, 2011–2017, *n* = 584	Bulgaria, 2018–2021, *n* = 216	Bulgaria 2022, *n* = 74
	Number/Percent *	Number/Percent *	Number/Percent *
*M. abscessus*	11/1.9	11/5.1	2/2.7
*M. chelonae*	47/8	17/7.9	2/2.7
*M. avium complex*	94/16.1	68/31.5	33/44.6
*M. celatum, M. shimodei*	2		1
*M. fortuitum*	62/10.6	23/10.6	4/5.4
*M. gordonae*	102/17.5	58/26.9	18/24.3
*M. mucogenicum complex*	10/1.7	8/3.7	3/4.1
*M. kansasii*	17/2.9		1
*M. lentiflavum*	89/15.2	4/1.9	4/5.4
*M. marinum*	1	1	1
*M. scrofulaceum*	2	1	1
*M. simiae*	1	1	1
*M. xenopi*	2	6/2.8	1
M. aurum complex **			1
*M. malmoense*		1/0.5	
*M. peregrinum*	19/3.3		1
*M. szulgai/intermedium*	1		
*M. terrae complex*			
*M. interjectum*		1	
*M. neoaurum*			
*M. shimoidei*		1	
*M. genavense/M. triplex*	1		
*Mycobacterium* spp.	123	15/6.9	
Total	584	216	74

* Percentages are shown for >1.5%; ** One *M. aurum* complex isolate was assigned to *M. bulgaricum* sp. nov. Data on 2011–2017 and 2018–2021 were published by Atanassova [32] and Bachiyska et al. [31], respectively.

**Table 2:** Identification of NTM species using Myco-biochip and target sequencing in the studied collection from Bulgaria.

Complex	Species	Number of Isolates
*M. chelonae-abscessus*	*M* *. chelonae*	2
*M. chelonae-abscessus*	*M. abscessus*	2
*M. aurum*	*M. bulgaricum* sp. nov.	1
*M. fortuitum*	*M. peregrinum*	1
*M. fortuitum*	*M. fortuitum*	2
*M. neoaurum*	*M. neoaurum*	1
*M. mucogenicum*	*M. phocaicum*	3
*M. terrae*	*M. hiberniae*	2
*M. terrae*	*M.* sp. GF74	1
*M. terrae*	*M. kumamotonense*	3
*M. shimodei*	*M. celatum*	1
*M. xenopi*	*M. xenopi*	1
*M. asi* *aticum-gordonae*	*M. gordonae*	2
*M. asi* *aticum-gordonae*	*M. paragordonae*	2
*M. marinum*	*M. marinum*	1
*M. gastri-kansasii*	*M. kansasii*	1
*M. lentiflavum*	*M. lentiflavum*	3
*M. lentiflavum*	*M. simiae*	1
*M. avium*	*M. timonense*	2
*M. avium*	*M. intracellulare*	5
*M. avium*	*M. avium*	10
*M. avium*	*M.* sp. GF28	1
Total samples		48
Total species identified		22

**Table 3:** Distribution of NTM species complexes in patients by gender.

NTM Complex	Male (*n* = 17)	Female (*n* = 29)	TOTAL (*n* = 46) *
*M. avium*	4	13	17
*M. fortuitum*	3		3
*M. terrae*	3	3	6
*M. asiaticum-gordonae*	1	3	4
*M. chelonae-abscessus*	1	3	4
*M. lentiflavum*	1	3	4
*M. marinum*	1		1
*M. mucogenicum*	1	2	2
*M. shimodei*	1		1
*M. xenopi*	1		1
M. aurum **		1	1
*M. kansasii*		1	1
*M. neoaurum*		1	1

NTM—nontuberculous mycobacteria. * Information was available for 46 of 48 patients. Species assignment was based on the final corrected species information in Table 1. ** One *M. aurum* complex isolate was assigned to *M. bulgaricum* sp. nov.

**Table 4:** Distribution of NTM species complexes in age groups of patients.

NTM Complex	16–49 Years Old (*n* = 7)	50–59 Years Old (*n* = 6)	>59 Years Old (*n* = 31)	Total (*n* = 44) *
*M. avium*	2	3	12	17
*M. fortuitum*	1		2	3
*M. terrae*	1	2	2	5
*M. asiaticum-gordonae*		1	3	4
*M. chelonae-abscessus*			4	4
*M. lentiflavum*	2		1	3
*M. marinum*	1			1
*M. mucogenicum*			2	2
*M. shimodei*			1	1
*M. xenopi*			1	1
M. aurum **			1	1
*M. kansasii*			1	1
*M. neoaurum*			1	1

NTM—nontuberculous mycobacteria. * Information was available for 44 of 48 patients. Species assignment was based on the final corrected species information in Table 1. ** One *M. aurum* complex isolate was assigned to *M. bulgaricum* sp. nov.

**Table 5:** Distribution of NTM complexes in provinces of Bulgaria based on the final corrected information obtained by Myco-biochip and sequencing of 40 samples from the year 2022.

Region	NTM Complex	Number of Isolates
Sofia	*M. avium*	11
Sofia	*M. chelonae-abscessus*	4
Sofia	*M. lentiflavum*	3
Sofia	*M. fortuitum*	2
Sofia	*M. asiaticum-gordonae*	1
Sofia	*M. aurum* *	1
Sofia	*M. kansasii*	1
Sofia	*M. mucogenicum*	1
Sofia	*M. xenopi*	1
Plovdiv	*M. asiaticum-gordonae*	3
Plovdiv	*M. avium*	3
Plovdiv	*M. mucogenicum*	2
Plovdiv	*M. fortuitum*	1
Pleven	*M. avium*	2
Pleven	*M. marinum*	1
Burgas	*M. shimodei*	1
Dobrich	*M. avium*	1
Kustendil	*M. avium*	1
Yambol	*M. lentiflavum*	1

NTM—nontuberculous mycobacteria. * One M. aurum complex isolate was assigned to *M. bulgaricum* sp. nov.

**Table 6:** Distribution of NTM species in Bulgaria and its neighbor countries.

Species *	Bulgaria, 2011–2022 *n* = 874	Serbia, 2009–2016, *n* = 85	Greece, 1990–2013, *n* = 71	Turkey, 2004–2009, (**) *n* = 38	Turkey, 2004–2009, (***), *n* = 39
	Number/Percent	Number/Percent	Number/Percent	Number/Percent	Number/Percent
*M. abscessus*	24/2.7	8/9.4	1	5/13.2	2/5.1
*M. chelonae*	66/7.6	3/3.5	5/7		3/7.7
*M. avium* complex	195/22.3	25/29.4	46/64.8	14/36.8	2/5.1
*M. celatum*, *M. shimodei*	3		1		
*M. fortuitum*	89/10.2	4/4.7	2/2.8	4/10.5	7/17.9
*M. gordonae*	178/20.4	5/5.9			17/43.6
*M. mucogenicum* complex	22/2.5				
*M. kansasii*	18/2.1	15/17.6	9/12.7	6/15.8	1/2.6
*M. lentiflavum*	97/11.1		1		2/5.1
*M. marinum*	3				
*M. scrofulaceum*	3			1/2.6	1/2.6
*M. simiae*	3			4/10.5	2/5.1
*M. xenopi*	9	20/23.5	2/2.8		
*M. aurum* complex ****	2				
*M. malmoense*	1		4/5.6		
*M. peregrinum*	20/2.3	5/5.9			
*M. szulgai/intermedium*	1			3/7.9	
*M. terrae complex*	6				
*M. interjectum*	1				
*M. neoaurum*	1				
*M. shimoidei*	1				
*M. genavense/M. triplex*	1				
*Mycobacterium* spp.	130/14.9			1/2.6	2/5.1
Total	874	85	71	38	39

* Data are shown for separate species except for *M. avium* complex and *M. terrae* complex. Data on Serbia, Greece, and Turkey were previously published [80,81,82]; ** For Turkey, “suspected” cases also refer to isolates designated as “causative and contaminant”; *** For Turkey, strictly “contaminant” cases; **** One *M. aurum* complex isolate from Bulgaria was assigned to *M. bulgaricum* sp. nov.

## Data Availability

Genome data were deposited to NCBI SRA and Genome databases (PRJNA1138261).

## Supplementary Materials

The following supporting information can be downloaded at: https://www.mdpi.com/article/10.3390/ijms251910434/s1.
