# Supplementary material for: The Intriguing Pattern of Nontuberculous Mycobacteria in Bulgaria and Description of Mycobacterium bulgaricum sp. nov
Source: Int J Mol Sci. 2024 Sep 27;25(19):10434. doi: 10.3390/ijms251910434 (PMC11476446; doi:10.3390/ijms251910434)
Supplement: Supplementary file 1 [file ijms-25-10434-s001.zip › ijms-3217390-supplementary.pdf]

Supplementary Table S1. Characterization of 48 NTM isolates from Bulgaria: patient information and molecular species identification

Sample ID	year	Age	Sex	Region	source	ZN	Hain result	Biochip result	*gyrB* sequencing alignment*	WGS closest species	Complex**
1	2022	67	f	Sofia	sputum	1+	*M. abscessus*	*M. abscessus*			*M. chelonae-abscessus*
2	2022	70	f	Sofia	sputum	(-)	*M. abscessus*	*M. abscessus*			*M. chelonae-abscessus*
3	2022	57	f	Kustendil	sputum	(-)	*M. avium*	*M. avium*			*M. avium*
4	2022	81	f	Sofia	sputum	1+	*M. avium*	*M. avium*			*M. avium*
5	2022	73	m	Plovdiv	sputum	3+	*M. avium*	*M. avium*			*M. avium*
6	2022	65	m	Burgas	sputum	(-)	*M. celatum*	*M. celatum*			*M. shimodei*
7	2022	60	f	Sofia	sputum	(-)	*M. chelonae*	*M. chelonae*	*M. chelonae* [SAMN09770287] (306-5-0)		*M. chelonae-abscessus*
8	2022	61	f	Sofia	BAL	(-)	*M. chimaera*	*M. intracellulare*	*M. intracellulare subsp. chimaera* [SAMN06111444] (322-0-0)		*M. avium*
9	2022	60	m	Sofia	sputum	(-)	*M. fortuitum*	*M. fortuitum*			*M. fortuitum*
10	2022	53	f	Plovdiv	sputum	(8 AFB)	*M. gordonae*	*M. gordonae*			*M. asiaticum-gordonae*
11	2022	59	f	Dobrich	sputum	(-)	*M. intracellulare*	*M. intracellulare*			*M. avium*
12	2022	54	f	Pleven	sputum	(8 AFB)	*M. intracellulare*	*M. intracellulare*			*M. avium*
13	2022	74	f	Sofia	sputum	1+	*M. kansasii*	*M. kansasii*			*M. kansasii*
14	2022	69	f	Sofia	BAL	(-)	*M. lentiflavum*	*M. lentiflavum*			*M. lentiflavum*
15	2022	16	f	Yambol	gastric lavage	(-)	*M. lentiflavum*	*M. lentiflavum*			*M. lentiflavum*
16	2022	47	m	Pleven	wound secretion	(-)	*M. marinum*	*M. marinum*			*M. marinum*
17	2022	82	f	Sofia	BAL	(-)	*M. mucogenicum*	*M. mucogenicum*	*M. phocaicum* [SAMD00117553] (674-20-0)		*M. mucogenicum*
18	2022	71	f	Plovdiv	sputum	(-)	*M. scrofulaceum*	*M. mucogenicum*	*M. phocaicum*		discrepant

							(M. scrofulaceum complex)	*(M. mucogenicum complex)*	[SAMN04634180] (674-3-0)	
19	2022		f	Sofia	sputum	(-)	*M. simiae*	*M. simiae*		*M. lentiflavum*
20	2022	78	m	Sofia	sputum	(-)	*M. xenopi*	*M. xenopi*		*M. xenopi*
21	2022	29	m	Sofia	no data	(-)	*M. avium*	*M. avium*		*M. avium*
22	2022	76	f	Sofia	BAL	(-)	*M. avium*	*M. avium*		*M. avium*
23	2022	61	f	Pleven	sputum	(-)	*M. avium*	*M. avium*		*M. avium*
24	2022	71	f	Sofia	sputum	(-)	*M. avium*	*M. avium*		*M. avium*
25	2022	35	f	Sofia	BAL	(-)	*M. avium*	*M. avium*		*M. avium*
26	2022	89	m	Sofia	sputum	(-)	*M. avium*	*M. avium*		*M. avium*
27	2022	65	f	Sofia	sputum	(-)	*M. avium*	*M. avium*		*M. avium*
28	2022	77	f	Plovdiv	sputum	(7 AFB)	*M. chimaera*	*M. marseillense*	*M. marseillense* [SAMN06064246] (282-0-0)	*M. avium*
29	2022	30	m	Sofia	no data	(-)	*M. fortuitum*	*M. septicum /peregrinum*	*M. peregrinum*	*M. fortuitum*
30	2022	69	m	Plovdiv	sputum	(-)	*M. fortuitum*	*M. fortuitum*		*M. fortuitum*
31	2022	67	f	Plovdiv	sputum	(-)	*M. gordonae*	*M. paragordonae*	*M. paragordonae* [SAMN13318592] (285-5-0)	*M. asiaticum-gordonae*
32	2022	69	m	Plovdiv	sputum	1+	*M. gordonae*	*M. paragordonae*	*M. paragordonae* [SAMN13318592] (285-5-0)	*M. asiaticum-gordonae*
33	2022	76	f	Sofia	BAL	(-)	*M. gordonae*	*M. gordonae*		*M. asiaticum-gordonae*
34	2022			Sofia	BAL	(-)	*M. intracellulare*	*M. intracellulare*		*M. avium*
35	2022	76	f	Sofia	BAL	(-)	*M. intracellulare*	*M. intracellulare*		*M. avium*
36	2022	69	f	Plovdiv	sputum	1+	*M. intracellulare*	[New profile 1]	*M.* sp. GF28 [SAMN09476219] (306-0-0)	*M. avium*
37	2022	65	m	Sofia	sputum	(-)	*M. intracellulare*	*M. marseillense*	*M. marseillense* [SAMN06064246] (285-0-0)	*M. avium*
38	2022	45	m	Sofia	sputum	(-)	*M. lentiflavum*	*M. lentiflavum*		*M. lentiflavum*
39	2022	76	m	Sofia	sputum	(-)	*M. chelonae*	*M. chelonae*		*M. chelonae-abscessus*
40	2022	69	m	Plovdiv	sputum	(-)	*M. mucogenicum*	*M. mucogenicum*	*M. phocaicum*	*M. mucogenicum*

									[SAMD0011755]<br>(674-20-0)		
41	2018	81	f	Lovech	sputum	(-)	*Mycobacterium*	*M. neoaurum*	*M. neoaurum*<br>[SAMN18915540]<br>(306-3-0)		*M. neoaurum*
42	2018	57	f	Plovdiv	sputum	(-)	*Mycobacterium*	*M. kumamotonense*	*M. kumamotonense*<br>[SAMN06064243]<br>(274-0-0)		*M. terrae*
43	2018	ND	f	Montana	sputum	(-)	*Mycobacterium*	[New profile 2]	*M. engbaekii*<br>[SAMN04216923]<br>(306-0-0)		*M. terrae*
44	2018	39	f	Haskovo	sputum	(+)	*Mycobacterium*	[New profile 2]	*M. engbaeki*<br>[SAMN04216923]<br>(306-0-0)		*M. terrae*
45	2019	78	m	Varna	sputum	no data	*Mycobacterium*	*M. senuense*	*M. senuensis*<br>[SAMN09476217]<br>(306-9-0)	*M.* sp. GF74<br>ANI=97,5%	*M. terrae*
46	2020	58	m	Varna	sputum	no data	*Mycobacterium*	*M. kumamotonense*	*M. kumamotonensis*<br>[SAMN03793173]<br>(306-0-0)		*M. terrae*
47	2020	64	m	Varna	sputum	no data	*Mycobacterium*	*M. kumamotonense*	*M. kumamotonensis*<br>[SAMN13905685]<br>(306-1-0)		*M. terrae*
49	2022	68	f	Sofia	sputum	(-)	*Mycobacterium*	*M. iranicum*	MC-434 (100%)<br>*M. goodi*<br>[SAMN07828250]<br>(305-27-0)	*M. iranicum*<br>ANI = 87%<br>*M. bulgaricum*<br>sp. nov.	*M. aurum*

BAL – bronchoalveolar lavae. * NCBI BioSample accessions of best hits are shown in square brackets; alignments matches, mismatches, and gaps are shown in round brakets.

** In green: according to Wengenack et al. (2024)

In brown: according to Zimenkov et al. (unpublished)
